# Early implementation learnings on acceptability and feasibility of “V”: a multi-level PrEP intervention designed with and for adolescent girls and young women in Zimbabwe

**DOI:** 10.1186/s12961-023-01040-3

**Published:** 2023-10-02

**Authors:** Thenjiwe Sisimayi, Definate Nhamo, Kumbirai Chatora, Gwendoline Chapwanya, Tinovonga Mawoyo, Getrude Ncube, Cal Bruns, Emily L. Harris, Katharine D. Shelley

**Affiliations:** 1 Harare, Zimbabwe; 2 Pangaea Zimbabwe AIDS Trust (PZAT), 27 Rowland Square, Milton Park, Harare, Zimbabwe; 3 Population Services International (PSI), 45 Piers Road, Sam Levy’s Village Block C, Harare, Zimbabwe; 4 Ministry of Health and Child Care (MoHCC), 4th Floor Kaguvi Building, Harare, Zimbabwe; 5 Matchboxology, 70, 7th Avenue, Parktown North, Johannesburg, 2193 South Africa; 6 United States Agency for International Development (USAID), Office of HIV/AIDS, Washington, DC USA; 7 PATH, Primary Health Care, 2201 Westlake Ave Suite 200, Seattle, WA 98121 USA

**Keywords:** PrEP, Zimbabwe, Adolescent girls, Feasibility, Acceptability

## Abstract

**Introduction:**

Adolescent girls and young women (AGYW) remain disproportionately affected by HIV in Zimbabwe. Several HIV prevention options are available, including oral tenofovir-based pre‐exposure prophylaxis (PrEP), however AGYW face unique barriers to PrEP uptake and continuation and novel approaches are therefore needed to empower AGYW to use PrEP. The objective of this study was to characterize early learnings from implementing a multi-level intervention consisting of fashionable branding (including a “V Starter Kit”), service integration, and peer education and support throughout a young woman's journey using oral PrEP across four phases of implementation, from creating demand, preparing for PrEP, initiation of PrEP, and adherence to PrEP.

**Methods:**

A mixed methods implementation research study was undertaken, including site observations and interviews to explore the acceptability of “V” and its relevance to target users, as well as the feasibility of integrating “V” with existing service delivery models. Interviews (*n* = 46) were conducted with healthcare workers, Brand Ambassadors, and young women purposively sampled from four implementation sites. Interview data was analyzed thematically using the framework method for qualitative data management and analysis. Project budgets and invoices were used to compile unit cost and procurement data for all “V” materials.

**Results:**

“V” was acceptable to providers and young women due to attractive branding coupled with factual and thought-provoking messaging, establishing “a girl code” for discussing PrEP, and addressing a gap in communications materials. “V” was also feasible to integrate into routine service provision and outreach, alongside other services targeting AGYW. Cost for the “V” branded materials ranked most essential—FAQ insert, pill case, makeup bag, reminder sticker—were $7.61 per AGYW initiated on PrEP.

**Conclusion:**

“V” is a novel approach that is an acceptable and feasible multi-level intervention to improve PrEP access, uptake, and continuation among AGYW, which works through empowering AGYW to take control of their HIV prevention needs. In considering “V” for scale up in Zimbabwe, higher volume procurement and a customized lighter package of “V” materials, while still retaining V’s core approach, should be explored.

**Supplementary Information:**

The online version contains supplementary material available at 10.1186/s12961-023-01040-3.

## Introduction

Adolescent girls and young women (AGYW) in Eastern and Southern Africa face a disproportionate and persistent HIV infection risk. AGYW represent 10% of the population, yet, account for 26% of new infections regionally [1–3]. Programmatic investments and scientific advancements have led to an expanding HIV prevention toolkit ranging from abstinence, partner reduction, male and female condoms, knowing your partner’s HIV status, antiretroviral therapy for HIV-positive partners, with novel biomedical products in the research and development pipeline [4]. Yet, the expanding set of options is matched by the recognition that AGYW’s agency remains constrained by complex personal, social, and structural barriers [5–7]. AGYW’s ability to select, access and adhere to the HIV prevention approach that is right for their unique lifestyle is influenced by a constellation of factors such as their personal perceptions of HIV risk and illness, partnership power dynamics and gender norms, and access to education and financial opportunities [8, 9].

Since 2015, the WHO has recommended the once-a-day pill known as oral pre-exposure prophylaxis (referred to commonly as “oral PrEP”) given its efficacy as an additional prevention option for individual’s at high-risk of HIV acquisition, including for AGYW [10]. In Zimbabwe, phased roll-out of oral PrEP began in 2015, with more widespread access to PrEP the focus of a 2-year national implementation plan [3, 11]. In December 2021, in commemoration of World AIDS Day, the President’s Emergency Plan for AIDS Relief (PEPFAR) announced its latest global program results: 2.9 million AGYW reached with comprehensive HIV prevention services; and, one million new clients enrolled on oral PrEP [12]. The evidence since oral PrEP’s introduction demonstrates its effectiveness as an addition to the HIV prevention toolkit, if AGYW can access and use it as instructed [13].

To continue the rapid progress since 2015, and to ensure oral PrEP’s continued scale-up is not constrained by the unique barriers that AGYW encounter, the global health community is increasingly turning to human-centered design (HCD)^1^ for co-created, custom-fit solutions to increase the market demand for and programmatic innovations to deliver oral PrEP [14–16]. Multiple communities of practice supporting the strategic integration of HCD within global health have defined how design can serve as a complementary method for global health practitioners seeking innovative solutions [17–19]. Alongside a growing evidence base from TB, malaria and across the HIV continuum of care, design has been described as an “essential medicine” to address complex global health challenges through people-centered and interdisciplinary efforts. For oral PrEP, design offers new approaches for gathering user insights, and for turning these insights into tangible solutions for iterative testing and implementation [20–24].

## Methods

### Program description

**Fig. 1:**
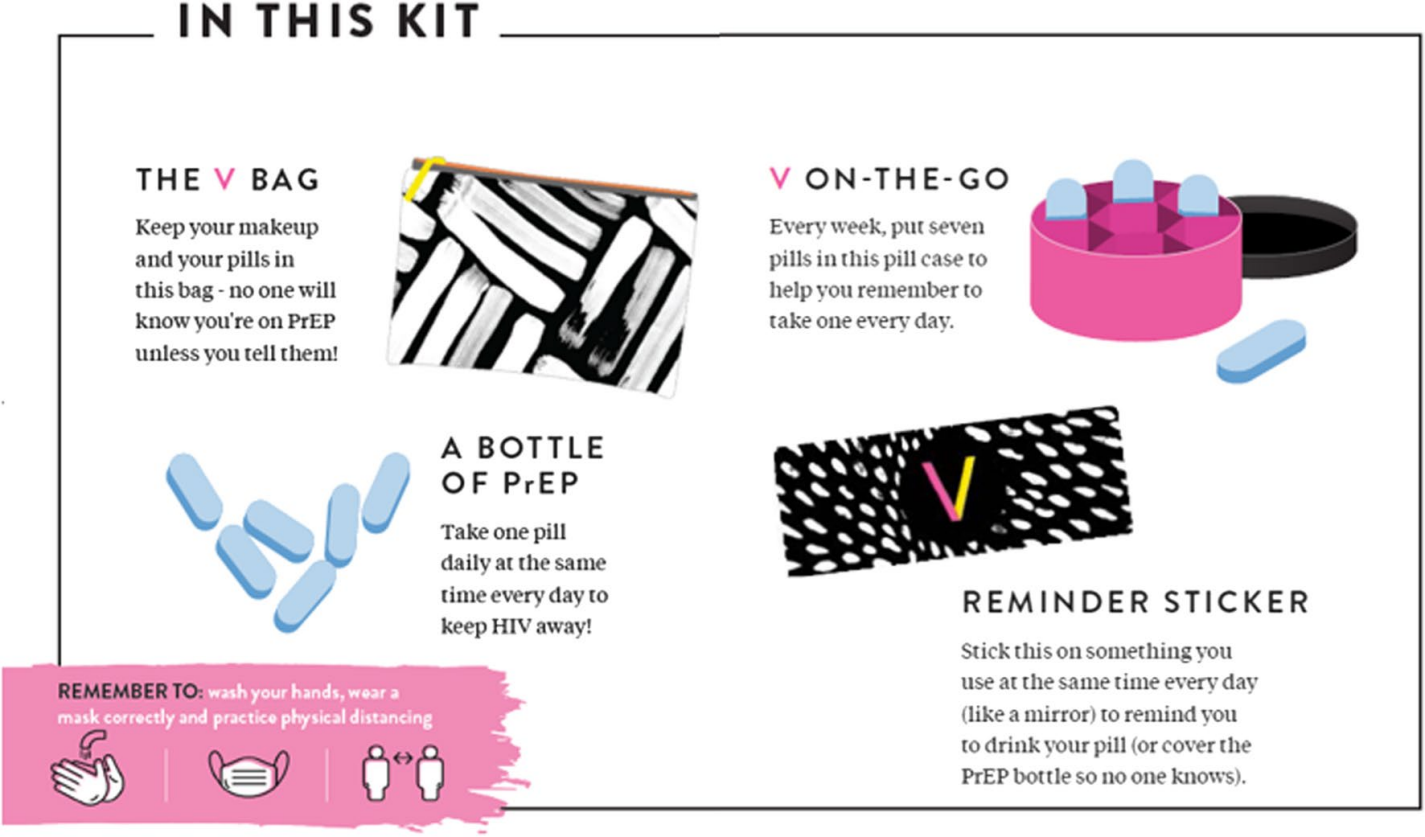
V supports AGYW along their PrEP journey by creating an exciting and empowering first experience with PrEP through a “V Starter Kit” delivered at PrEP initiation by supportive health care workers

Aligned with the increasing investment in HCD to meet the global and regional need for accelerating oral PrEP access, “V” is a brand and service delivery strategy that was designed to increase oral PrEP uptake and continuation among AGYW by shifting the messaging from “don’t get HIV” to “empower yourself.” Following the principles of user-driven design, young women and healthcare providers in South Africa informed V’s development, resulting in four programmatic pillars—Create Demand, Prep for PrEP, Initiation, and Continuation—and associated materials to support demand creation, implementation, outreach, and peer support [25]. The materials include posters, an educational pamphlet, flipchart, healthcare worker training manual, “V” Starter Kit (makeup bag, pill case, FAQ booklet, reminder sticker and box for pill bottle, Fig. 1), Brand Ambassador PrEP toolkit, and T-shirts—all of which help to enable a PrEP service provider to reframe a young women’s PrEP user experience, moving from “demand” for a product to “desire” for a product. The “V” branded materials help support the overall service delivery strategy and messaging to empower women to take control of their health.

### Study design and intervention components

**Fig. 2:**
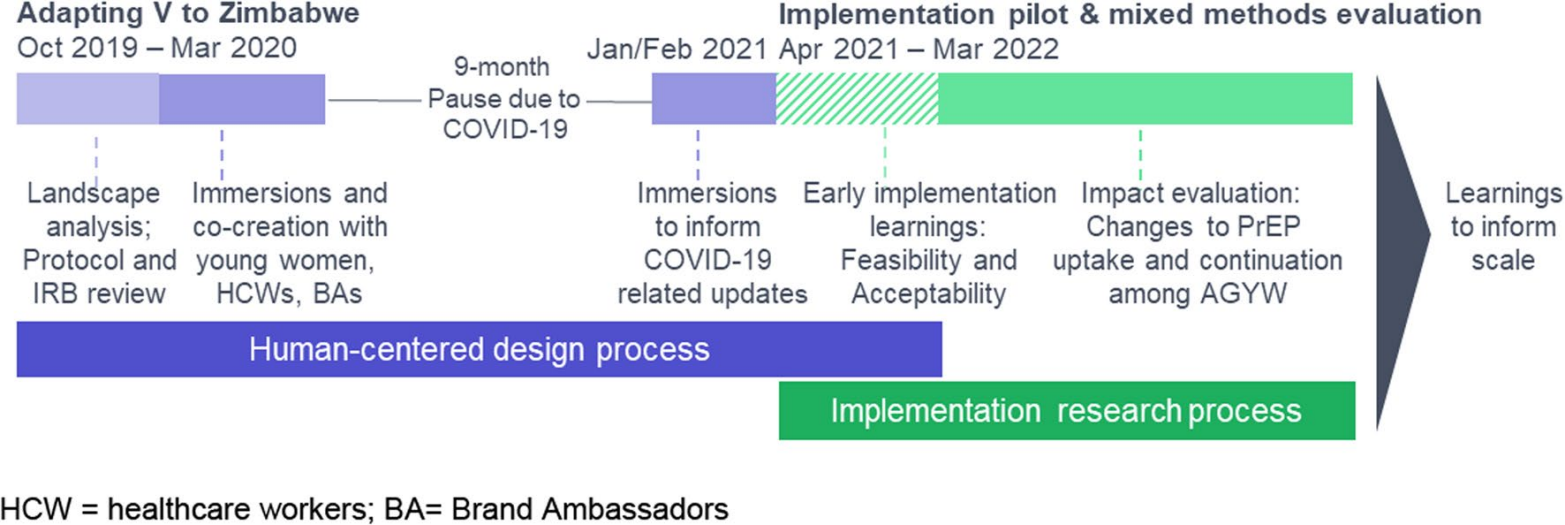
Timeline for design, implementation, and evaluation activities. *HCW*  healthcare workers, *BA*  Brand Ambassadors

A mixed methods study was undertaken from 2019 to 2022, including a HCD process to inform the overall intervention design (blue) and implementation research to answer key learning questions (green) (Fig. 2).

In 2019, the EngageDesign consortium—including Matchboxology and PATH—partnered with Population Services International Zimbabwe (PSI), Pangaea Zimbabwe AIDS Trust (PZAT), and the Zimbabwe Ministry of Health and Child Care (MoHCC) to adapt the “V” brand and approach to local contexts in Zimbabwe, including designing implementation strategies to integrate “V” within existing healthcare delivery platforms with the aim of increasing PrEP uptake and continuation among AGYW. The end-to-end HCD process and adaptations of “V” materials to Zimbabwe is detailed by Harris et al. [26]. In brief, from October to December 2019, we carried out an initial landscape market analysis and consultations with stakeholders involved in PrEP service delivery in Zimbabwe, developed a study protocol and data collection tools, and underwent ethics review and approval. Four district settings—Bulawayo, Gweru, Chipinge, Chitungwiza—were selected for adapting and implementing “V”, all of which offer services in static health facilities and through outreach approaches and represent a variety of geographic and demographic settings. The New Start Centers (NSC) managed by PSI differ slightly in terms of their reach and location with Bulawayo (Bambanani NSC) offering services in an urban locale, Gweru NSC covering both urban and peri-urban communities, while Chipinge NSC predominantly services rural communities. An adolescent friendly space (the SHAZ! Hub) is located within a private hospital and is managed by PZAT as a Private–Public Partnership with Zimbabwe’s MoHCC and serves youth in the peri-urban community of Chitungwiza District. In both PSI and PZAT sites, Brand Ambassadors bridge connections between communities and health facilities through serving as PrEP champions and peer educators.

In January and February 2020, we facilitated HCD immersions with healthcare workers, young women, brand ambassadors, and influencers in four districts and held co-creation workshops for adapting “V” materials and implementation strategies to integrate “V” within existing services delivery models. The project was originally intended to begin pilot implementation in April 2020 but was delayed due to the COVID-19 pandemic. In early 2021, additional immersions were conducted to inform updates to “V” materials to accommodate changes in service delivery resulting from COVID-19, for example including messaging related to personal protective equipment and social distancing.

Due to COVID-19 restrictions, training to prepare for the “V” launch was conducted virtually. A total of 30 healthcare workers and 28 Brand Ambassadors across the four sites participated in “V” training from 7–9 April 2021. The training focused on the objectives of the pilot, what the “V” pillars and materials consisted of and how to integrate them into implementation contexts and scenarios. The training approach included a variety of presentations on the “V” materials, role play scenarios on utilizing the “V” materials and practicing key messaging, and question and answer discussion time. Participants facilitated the role clarification training section as a way of taking ownership and demonstrating their understanding of how “V” was intended to be implemented. Healthcare workers who participated in the virtual training subsequently convened an onsite staff orientation within a week to ensure site readiness for the “V” implementation launch. An implementation guide/playbook was used to guide the orientation of all staff at the sites and remains a reference material for ongoing implementation. The four sites began “V” implementation on 15 April, 2021.

### Learning questions

Here we report findings from multiple types of data—site monitoring calls and observations, in-depth interviews, routine programmatic service delivery indicators, and project budgets and invoices—collected to answer the following learning questions around early implementation experiences, including adaptations to inform ongoing implementation and scale up, including:

- Is “V” acceptable to healthcare workers, Brand Ambassadors, and AGYW? Why or why not?
- Is “V” feasible for healthcare workers and Brand Ambassadors to introduce and implement? Why or why not?
- What needs to be adapted about “V” implementation going forward?
- What are the key cost considerations for implementing “V” in clinics already offering PrEP services to AGYW?

Outcomes data on PrEP uptake and continuation will be reported in a subsequent publication following a full year of “V” implementation.

### Sampling and qualitative data collection

Two rounds of site monitoring calls (26–30 April 2021 and 30 June–2 July 2021) were conducted with each of the four facility-based Site Managers utilizing a site monitoring checklist tool to obtain information regarding PrEP service provision at the facility and via outreach services, with an emphasis on understanding implementation fidelity and context (Additional file 1). Site monitoring calls included video walkthroughs of the clinic space to review how “V” poster materials and flip charts were set up, to explain how “V” was integrated into existing clinic flows, and to troubleshoot challenges.

After each round of site monitoring, in-depth interviews (10–14 May 2021 and 13–26 July 2021) were conducted using a semi-structured interview guide designed to elicit perceptions about the acceptability and feasibility of “V”, whether any further adaptations were needed to “V” materials or the implementation approaches (during the remaining pilot period and/or for broader scale up considerations), and to document any unintended consequences of the “V” intervention (Additional file 2). Standard definitions of *acceptability* and *feasibility* from the implementation research literature [27] were integrated into interview questions. In line with COVID-19 precautions, all data was collected virtually via phone, Whatsapp, or Zoom calls by the project’s co-Principal Investigator based in Harare, Zimbabwe. Three types of respondents were purposively sampled from the four project sites: healthcare workers involved in implementing “V” at facilities and outreach sites, Brand Ambassadors involved in engaging AGYW and creating demand for “V”, and young women ages 18–24 who were offered PrEP at the four project sites (Table 1). Young women were purposively recruited by healthcare workers based on their availability and willingness to participate in the study, and to ensure a mix of young women’s experiences including (1) young women who initiated PrEP after “V” launched; (2) young women who had discontinued PrEP previously, but re-initiated PrEP after exposure to "V"; and (3) non-PrEP users, including those that had either previously discontinued PrEP or had never used PrEP.

**Table 1:** Summary of total in-depth interviews and sampling approach for each respondent type

Respondent type	Interviews	Sampling approach from four implementing sites
Healthcare workers	10*	Purposively sampled, including healthcare workers involved in implementing V in each of the four locations
Brand Ambassadors	10^	Purposively sampled, including Brand Ambassadors involved in implementing V in each of the four locations
Young Women (18–24 years old)	26	Purposively sampled to ensure a variety of experiences, including current PrEP users (*n* = 19), former PrEP users who re-initiated PrEP after V launched (*n* = 2), PrEP defaulters (*n* = 4) and PrEP non-users (*n* = 1)
Total	46	

^*^7 unique respondents (1 healthcare worker at Bulawayo, Gweru, and Chitungwiza sites interviewed in both rounds)

^6 unique respondents (1 Brand Ambassador at each of the four sites was interviewed in both rounds)

All participants provided verbal informed consent to participate in the interviews and to be audio-recorded for transcription purposes. Interviews were conducted in English, Shona, or Ndebele depending on the preferences of the respondent. Brand Ambassadors and young women who participated in the interviews received US$10.00 each for data and airtime reimbursement. In total, 46 respondents were interviewed, including 9 healthcare workers, 10 Brand Ambassadors, and 27 young women (including 4 healthcare workers and 2 Brand Ambassadors interviewed at both time points in May and July) (Table 1). Interviews with young women generally lasted 20–45 min, whereas interviews with healthcare workers and Brand Ambassadors lasted approximately 60–90 min. Some interviews extended beyond one hour due to dropped calls from unstable network connectivity issues.

Qualitative site monitoring and interview data were supplemented by review of routine PrEP indicators, including the number of AGYW enrolled on PrEP per month and the number of AGYW on PrEP returning for a refill after one month, to examine early trends in outcomes relative to the pre-V baseline period and to triangulate with perceptions and experiences shared by interview respondents.

### Qualitative data management and analysis

The interviewer transcribed and summarized notes and illustrative quotes and organized the data in a Microsoft Excel-based matrix, segmented by respondent type and district, to enable data comparisons for each thematic area in line with the framework method for qualitative data management and analysis [28] and rapid analytical approaches for applied qualitative research [29]. Qualitative data were organized in a framework according to the overarching themes identified a priori (reasons for acceptability and feasibility; types of implementation challenges; unintended consequences; recommended adaptations for improvement). The data display matrices helped to chart and identify recurrent data patterns, emerging learnings, and gaps in the data requiring further exploration during the second round of interview data collection. The evaluation team met 1–2 times per week throughout the data collection period to discuss emerging learnings through triangulation of interview data, site monitoring data, and trends in PrEP uptake. The EngageDesign consortium also convened a series of monthly learning sessions with the implementers (PSI and PZAT) and the donor to share emerging evidence. Key findings are presented in the context of understanding the acceptability and feasibility of “V” and the unintended consequences identified during early implementation.

### Cost data and analysis

We reviewed project budgets and invoices to compile unit cost data and procurement quantities for all “V” materials used in the “V” pilot in Zimbabwe. Cost data is presented for the full package of “V” materials as well as for a subset of materials for AGYW deemed “most essential” by the interview respondents. Other cost considerations related to adapting the “V” materials to the Zimbabwean context are described qualitatively based on experiences from the EngageDesign consortium.

## Results

### Respondent characteristics

A total of 46 in-depth interviews were conducted with three respondent types sampled from across the four project sites including: 10 interviews with healthcare workers (7 unique respondents), 10 interviews with Brand Ambassadors (6 unique respondents), and 26 interviews with young women (Table 1). All healthcare workers and Brand Ambassadors interviewed had previously attended the training sessions at the launch of “V” and were involved in implementation of "V". Among the eight healthcare workers interviewed, qualifications included integrated HIV care nurses (*n* = 4), nurse counselor (*n* = 1), registered nurse clinician (*n* = 1), midwife (*n* = 1). Brand Ambassadors, all female, ranged in age from 23 to 27 (mean = 24.6 years), and included a mix of current or previous PrEP users (*n* = 4) and PrEP non-users (*n* = 2). The age of young women respondents ranged from 18 to 24 (mean = 21.1 years). Most young women interviewed reported current PrEP use at the time of interview (*n* = 21), including two who previously discontinued PrEP and reinitiated after “V” launched. Among current PrEP users interviewed, most were newly initiated after V’s launch and reported using PrEP for 1 month or less (*n* = 2), 2 months (*n* = 10), 3 months (*n* = 3), or 4 months (*n* = 2), meaning most had already returned after 30 days to pick up their first 3-month refill for PrEP; and a minority reported long term PrEP use of 2 years (*n* = 4). Among the five non-users interviewed, several reported discontinuing PrEP use (*n* = 4; three discontinued after two weeks of use, and 1 discontinued after three months) and one reported never having used PrEP.

### Acceptability of V

V resonated strongly with respondents for several reasons, including (1) attractive branding, (2) establishing a ‘girl code’ for PrEP, (3) factual and thought-provoking content, and (4) addressing an existing need.

#### Attractive branding

The “V” branding is attractive and in touch with current trends, including bright bold colors, stylish font and contextual images, while also being positively different. *“V ineka that, that, that!”* is slang that was commonly used by young women when describing “V”, which roughly translates as “V” is in a class of its own and cannot be described by words. Interviews with young women highlighted that being associated with “V” is desirable and motivating. For example:

“…The [V] starter-kit motivated me to take PrEP. You know, I already knew about PrEP because the BA [Brand Ambassador] had talked to me about it before, but it was stigmatizing and embarrassing to say PrEP and even to think about it because it was like ARVs for people who are on HIV treatment. So when I heard that it’s now “V” and has these slaying things I was fascinated and wanted it….my favorite pieces are the 7 day pill case and the “V” bag…” Young Woman

“…Yes, I took “V” because I wanted the beautiful bag and in the process that’s when I realized that what I needed was PrEP more than the bag. “V” protects my whole body…” Young Woman

The attractive branding and colors were also tied to feelings of ownership of a treasured personal asset. AGYW who would ordinarily not have a chance to own a make-up bag through the “V” starter kit were offered a gift to use to take care of oneself and keep safe from HIV. For example:

“…I have never owned a beautiful thing like a bag and something that looks so expensive and cool. It makes me feel vibrant. No-one can buy me that kind of bag and lip-gloss bottle. I feel proud to own it…” Young Woman

“…I’m now using the [V] starter kit accessories for swag. Like the bag I carry it along and keep my phone, money, ID, lipsticks and other small items that fit in it…” Young Woman

#### Establishing a ‘girl code’ for PrEP

“V” means PrEP and it became part of the ‘girl code’: “V” eliminates the need for using the term ‘PrEP’ and makes referring to PrEP easy and an open secret code for girls who are smart to slay and take care of themselves at the same time. For example, a young woman explained that “V is PrEP. “V” is HIV prevention and “V” is style”. Healthcare workers also reflected on how “V” helped establish common ground in speaking about PrEP in “code” that was easily understood by AGYW and providers alike. The “V” terminology promotes discretion while also helping to reduce young women’s concerns that PrEP is stigmatized as a pill associated with sex workers and men who have sex with men; AGYW who did not identify with sex work would not want to be associated with the PrEP pill. “V” also helped differentiate PrEP (prevention) from ART (treatment), which addressed another reason AGYW had avoided PrEP due to fear and stigma of being perceived as HIV-positive.

“…V is PrEP. We prefer to say that and that’s what everyone knows “V” to be although we know that PrEP is PrEP and “V” is a lubricant for taking PrEP, it is a spice. So “V” is shorter than PrEP so they say “V” V “V” not PrEP...” Brand Ambassador

"...Va Va Voom…many girls use it but mostly in a funny way. They use “V” mostly to mean ‘Peace’ and girl code…" Brand Ambassador

#### Factual and thought-provoking content

**Fig. 3:**
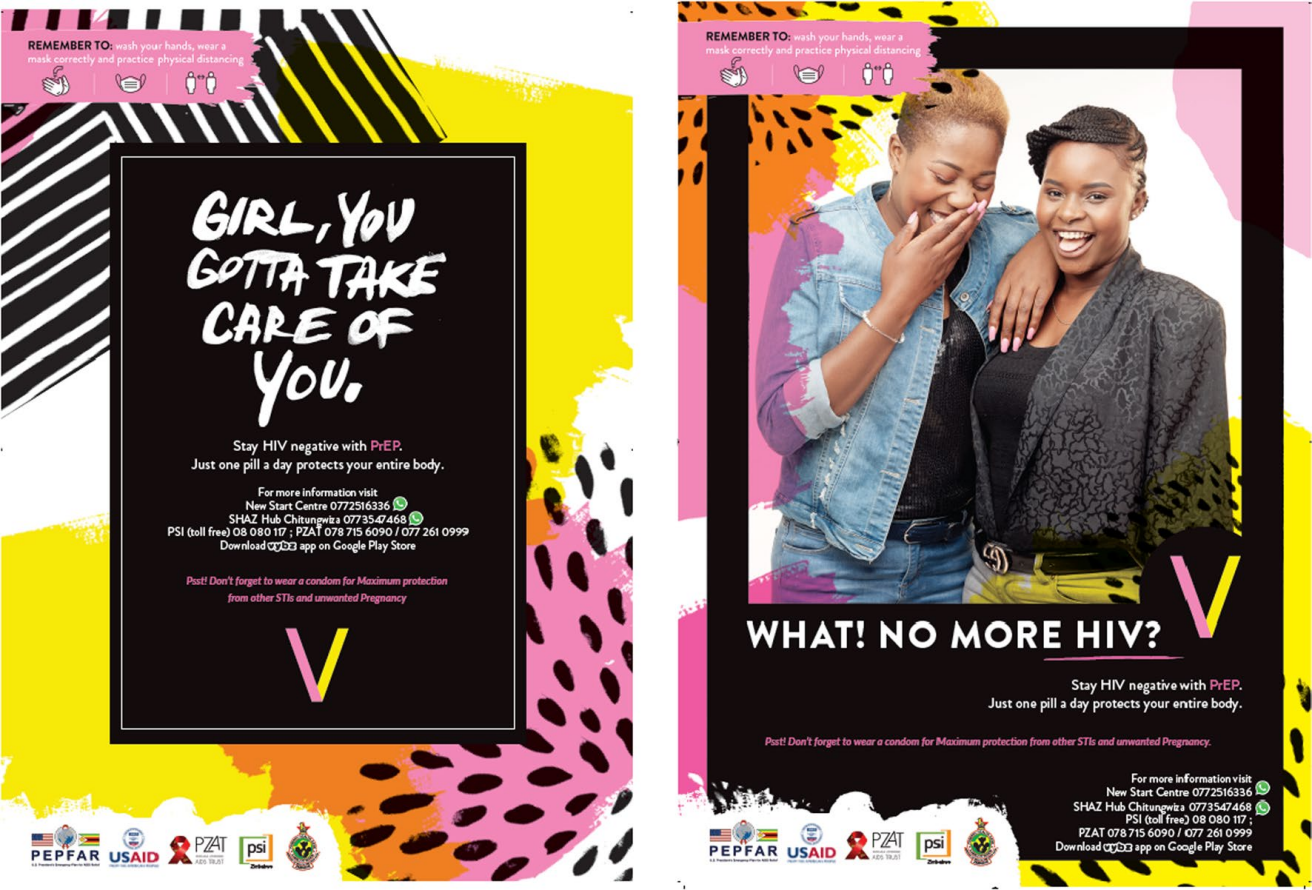
V posters that generated the most interest and curiosity among young women

The “V” materials include factual, concise, and thought-provoking content which prompts readers to inquire what the message is all about. Statements on the posters served a double purpose of sharing information and prompting curiosity on a subject of interest to anyone who is at risk of HIV. One Brand Ambassador shared that "… ‘*Girl you gotta take care of you*’ and ‘*No more HIV*’ are the two posters that cause discussions to become lively. They want to know what has happened to say there is no more HIV and they want to know how they can take care of themselves and that’s when “V” becomes a darling…” (Fig. 3). Similarly, another noted that:

“V is influencing uptake of PrEP in two ways. The first is that it gives more information. Messages like "No more HIV'' or "Some people will never get HIV" raise curiosity and they want to know, so they ask. Then the [V] Starter Kit influences the decision to take PrEP because then they learn that the pills can be taken as part of their routine, discreetly and they can choose to tell someone or not. They also get to know other girls on PrEP. The desire to own the “V” bag and pill case also attracts the girls to assess their risk and take PrEP.” Brand Ambassador

#### “V” was timely and addressed an existing need

Both healthcare workers and Brand Ambassadors lacked information, education, and communication (IEC) materials to assist in the drive to promote uptake and continuation of PrEP among AGYW. The launch of “V” bridged the gap and made reference materials available that complemented ongoing PrEP programming and demand generation efforts, including comprehensive information for personal risk assessment. Brand Ambassadors were equipped with the “V” Starter Kits which made engaging in dialogue with AGYW much easier, fancier and more authentic. Several elements of the “V” Starter Kit addressed barriers faced by AGYW, for example, the pill case and sticker addressed a problem of storage of pills and unwanted disclosure and fears of stigma. A young woman explained “…V is happiness and privacy. You take it quietly and that is good…” The FAQ guide contains rich information and clarifies PrEP as an HIV prevention medicine and not ART for people living with HIV. The “V” Starter Kit and FAQ guide brought convenience (make-up bag and pill case) and confidence (information from FAQ guide empowering through knowledge). The HCW flip chart eases the process of explaining issues to the clients where reference is required.

“…Since we started “V”, my audience with the girls has increased in numbers. Even those that did not want it before. Now I receive messages even at night from others asking for PrEP and those already on PrEP now asking if I can extend “V” and PrEP to their friends. I am now enjoying doing the community work…” Brand Ambassador

“…V is offering a discreet way of taking the pills. Because they knew about PrEP but when they think about the issues of privacy they ignored it because of lack of privacy and confidentiality. Before “V” came we used to have health education talks and they would come in numbers. You would see that they want PrEP but still don’t take it because they worried that other people will see them…” Healthcare Worker

The “V” materials also supported healthcare workers in using terms that are more familiar to AGYW, thereby finding a common starting point for a conversation around beauty and care issues which are considered a favorite topic for many of the women and girls. For example:

“…We are using the language in the posters like “Girl, you got to take care of you”. “V” has brought us to a common ground – we can talk about everything now and relate on that bag because all women and girls love to talk about beauty…” Healthcare Worker

### Feasibility of “V”

#### Integrating “V” within routine service delivery

Implementing “V” in a variety of settings including static facilities, outreach settings, and online fora, was considered feasible by healthcare workers and Brand Ambassadors. “V” was designed to easily fit into all the pillars of ART continuum of care (Prevention, Response, Care and Support, and Coordination) and providers reported ease in drawing on any of the “V” assets as a conversation starter with AGYW, either in-person or virtually/online. For example, online a Brand Ambassador could send a digital version of the poster or pictures of “V” assets to spark a discussion and increase online engagement among AGYW, while in person they could refer to the physical poster to discuss the message or open the pill case to ask what they think it can be used for. The “V” Starter Kit is portable so that Brand Ambassadors were able to carry it around and use it to engage the girls to explain PrEP and interest them to consider taking PrEP. For example:

“The entire “V” is a game changer for all healthcare workers because it is a conversation starter. They have allowed all the HCW and general hands or workers to talk about PrEP. Everyone in the site is able to speak about PrEP using “V” as the conversation starter.” Healthcare Worker

“Implementation has been good so far. We are initiating more girls on PrEP. Engaging the girls on PrEP has also improved since we now have the “V” materials to do it. Unlike previously when we were just talking without anything to show. It was harder to find girls to initiate on PrEP. There are some that we talked to before and showed no interest because of the stigma around the PrEP bottle. So now they are coming to take PrEP, they are commenting nice on “V” and they are saying that now they are motivated because they have somewhere to keep their PrEP and take it with some pride. They will not be stigmatized and can keep their status private.” Brand Ambassador

Interviews indicated that healthcare workers were very familiar with “V” and comfortable answering questions from AGYW, as well as questions from other clinic attendees who may ask what “V” all is about. Due to its ease of integration, healthcare providers talk about “V” to all eligible clients (women 15–24 years old) regardless of what other service the client sought, for example using sexual and reproductive health, family planning, menstrual hygiene, gender-based violence, and HIV testing as an entry point for introducing "V". However, COVID-19 restrictions meant that Brand Ambassadors sometimes needed to shorten their educational messaging to focus on PrEP, rather than first covering menstrual hygiene and family planning.

#### Communication channels

SMS reminders were designed to be sent once a week to AGYW clients newly enrolled on PrEP. Brand Ambassadors considered the guides for SMS reminders helpful, although not many SMS reminders were sent, in part because SMS data is more expensive compared to WhatsApp messaging. Brand Ambassadors asked AGYW accessing any of the HIV, family planning, and/or gender-based violence services from static and outreach sites if they would like to be added to the WhatsApp “Let’s Talk” groups for continued peer-based support, encouragement, reminders, updates regarding outreach services, and learning and sharing experiences with any of the products. Clients that opted into the WhatsApp groups, managed by the Brand Ambassadors, received reminders daily when chats came through. WhatsApp groups have served as a convening and supportive space for sharing information, building confidence, and asking questions about PrEP and V. For AGYW without access to phones, especially the younger adolescents and those living in rural Chipinge where internet and mobile phone network coverage is limited, these clients have to use other means to remember to take PrEP. Most choose to take PrEP at the same time they take their oral contraceptive pills.

### Challenges and unintended consequences

While “V” was feasible, early implementation experiences revealed several challenges and/or unintended consequences, including (1) unwanted disclosure, (2) substantial interest in “V” from beyond the AGYW target group, and (3) issues in adapting “V” parties to online engagements.

#### Unwanted disclosure

As the “V” brand became more and more visible in the community, the “V” makeup bags started to become a unique identifier for PrEP users, which increased the chances for unwanted disclosure of PrEP use and/or the potential for increased stigma. PrEP users reported being identified and having the PrEP use status being openly talked about in public spaces upon being seen carrying the makeup bag. For example, a young woman shared a recent interaction, “…Aaah, so you too are also on PrEP? I know that bag, my daughter got it also, she is on PrEP…” The implications are that the “V” makeup bag can reduce the discretion that “V” sought to offer AGYW on PrEP. Another closely linked issue regarding the “V” makeup bag was that it could attract attention from older men (“sugar daddies”) to target AGYW for unprotected sexual intercourse on the basis that they know these girls are HIV negative since they are on PrEP.

The text on the “V” pill box can also perpetuate unwanted disclosure as it specifies the contents of the box referring to HIV prevention pills and attracts increased attention from parents and/or partners because it looks like a perfume box. For example:

“...I blame the box for causing problems for me though. Without the box, he wouldn’t have seen it. Because the rest did not bother him. He said the box attracted him. It was standing out. He was curious who got me a perfume and he started suspecting me. Even after I showed him the booklet, he went on to say ‘so I am planning to go sleeping around that’s why I have taken PrEP’…” Young Woman

To minimize the risk of stigma or unwanted disclosure, possible recommendations put forward from the interviewees include offering the makeup bags in more assorted colors, as well as including more content that promotes self-efficacy for AGYW to make informed choices (such as negotiating condom use in combination with PrEP). For scale up, implementers could also consider whether to drop the “V” makeup bag and pill box altogether (see Adaptations below).

#### Interest in “V” beyond AGYW target group

Healthcare workers and Brand Ambassadors shared that adults currently on PrEP and ART felt that restricting “V” to AGYW was unfair as it denied them the “V” Starter Kit and the convenience it affords in supporting medicine adherence (specifically, the pill case). The problems associated with convenience and discretion of taking HIV prevention and treatment medicines are not unique to AGYW. In particular, the Brand Ambassadors had to manage backlash from older age groups that do not qualify for “V” assets, and in addition there were some perceptions that the Brand Ambassadors were responsible for promoting early sexual debut and encouraging secrecy between adolescents and their parents. Given these concerns, more consideration is needed on whether the whole “V” Starter Kit, or some components (the pill case, in particular) may be expanded to other PrEP and ART users. There is also a need to support Brand Ambassadors with additional messaging around why “V” was originally designed to target AGYW.

#### Adapting “V” parties to online engagements

In addition to integrating the “V” brand into existing service delivery strategies within clinics and through outreach events, Ambassador-led “V” parties were designed as in-person engagements with 10–15 AGYW to generate excitement around “V” and to answer questions about PrEP in a casual and fun environment with refreshments and music. After a few minutes for brief introductions and icebreakers, the discussion was designed to cover menstrual health (including offering menstrual cups to those who need them), family planning methods, and HIV prevention, including PrEP explained through using the “V” materials. At the end of “V” party, the participants can choose what types of referrals they are interested in for additional services at the clinic or outreach events. Those who agree will have their mobile numbers added into WhatsApp “Let’s Talk (Site)” Groups. However, due to COVID-19, “V” parties could not be convened in-person unless the size was reduced to five or fewer AGYW—which would not have been cost-effective in the context of programming targets. Switching to online “V” parties was possible in some settings, but raised several challenges as discussed below.

The online “V” parties varied from the original design in that they combined current and prospective PrEP users as well as non-users, and those who have discontinued PrEP, whereas the in-person “V” parties were originally designed for demand creation among non-users of PrEP. The online “V” parties were hosted over WhatsApp with approximately 12–15 AGYW attending each party, which lasted between 1–2 h. To kick-off the party, a Brand Ambassador posted a question in the WhatsApp chat and the party attendees would respond through voice notes or typed responses, which were sometimes posted by AGYW more than 20 min after the initial discussion question was framed. Although this approach could not fully mimic the real time conversational style achieved through an in-person event, qualitative interviews indicated that “V” party participants considered the exchange of information to be useful, especially testimonials from current PrEP users. In addition, there were several challenges to hosting virtual gatherings including: parties often took longer than expected; not everyone would be online at the same time; some participants were affected by network challenges or had power limitations; and recurrent costs associated with providing airtime for attendees. Since the initial set of “V” parties, PSI Brand Ambassadors have not continued to convene more online parties, in part due to cost considerations for airtime reimbursement, and have instead relied on online engagement via the WhatsApp groups.

PZAT was unable to conduct online “V” parties as they had already procured refreshments for the in-person parties and had no additional funding to facilitate the online parties; in addition, most of their clients do not have smartphones. Both implementing partners indicated that there is a need for some additional training for Brand Ambassadors to understand how to conduct and effectively facilitate virtual “V” parties, as emphasized the following quotes:

“The shift to using online has been tough for me. I really can't type everything like all the information in the way I would when I am talking physically with someone. I now spend more time addressing one person when I could use the same amount of time with more people. These days with network challenges, some messages delay delivering at all. I feel the “V” materials are better used physically.” Brand Ambassador

“The challenge is the network because everyone wants to post a voice note because typing would take a long time, and some can’t really express themselves with typing... We need training on “V” online party facilitation. We really don't know how to facilitate them and don't know which questions to ask first.” Brand Ambassador

### Adaptations to “V” materials

Data on acceptability and feasibility helped identify which of the “V” assets were deemed most ‘essential’ and ‘useful’, and whether and how the “V” materials could be further improved before wider scale up (Table 2). Suggested adaptations were generally minimal (e.g., color, size) given the materials had previously been adapted through a HCD approach [26]. However, the early implementation experiences suggested excluding some assets from scale up, either to address unintended consequences or to reduce recurrent costs by paring down the materials to only the most essential.

**Table 2:** Considerations for further "V" material adaptations based on early implementation

Materials	What	How to adapt	Comments
Makeup bag	Color	Consider multiple assorted bag colors	This is essential to minimize risk of stigma or unwanted disclosure
Educational pamphlet	Size	Reduce the size from A4 to A6 or Business Card Size	The A4 pamphlet attracts unwanted attention and needs to be resized to fit in a bag or pocket without having to fold it
FAQ booklet	Contact details	Include contact details for facilities offering PrEP and other support	The FAQ must include contact information (e.g., WhatsApp numbers) for support and PrEP service availability
Pill box		Consider excluding pill box from full implementation	The text on the pill box facilitated unwanted disclosure and defeats the purpose of discreet intent
Reminder sticker	Timing	Consider distribution frequency	With multi-month refills, some AGYW indicate wanting multiple stickers for each pill bottle. Note that although V did not promote use of stickers to cover the pill bottle, many AGYW used it for that purpose
HCW Manual	Content	Incorporate new PrEP guidelines as needed	Continue updating with new information to ensure content remains current (as needed)
HCW Flipchart	No adaptations	No adaptations	Difficult to use the flip chart in the context of social distancing measures
BA Toolkit	No adaptations	No adaptations	Essential for training purposes and as a reference source
Pill case	No adaptations	No adaptations	No adaptations
Posters	No adaptations	No adaptations	No adaptations
T-Shirts	No adaptations	No adaptations	No adaptations

### Cost of “V” materials

We identified four areas of cost considerations that are critical for adapting and implementing “V” within existing PrEP programs: Immersions and co-creation; adaptations to “V” materials to new contexts; training; and procurement of “V” materials/assets. The costs are highly variable depending on context, team availability, level of adaptations required, and package of “V” materials selected. Here we present only the costs of the “V” materials procured for this pilot, organized by start-up and recurrent costs of “V” materials needed at the clinic level (for use by healthcare workers and Brand Ambassadors), versus the per AGYW cost of materials for the “V” Starter Kit (Table 3). As noted above, some “V” materials were deemed ‘essential’ by AGYW, Brand Ambassadors or healthcare workers, while others were considered ‘nice to have’ and could be dropped in an expanded “V-lite” approach with fewer “V” materials but still retaining the essence of the “V” approach. Young women interviewed ranked the FAQ insert, the pill case, the makeup bag and the reminder sticker as most essential and useful, which sum to an approximate cost of $7.61 per AGYW initiated on PrEP. Additional materials highly rated for implementation of “V” include data/airtime for Brand Ambassadors, T-shirts, the educational flipchart, and the Brand Ambassador toolkit.

**Table 3:** Unit costs for "V" materials, organized by client facing, brand ambassadors, and AGYW

Level	V materials^#^	Unit	Budget and implementation notes
Clinic: Client facing	Educational flipcharts	$9.29	Budgeted 12–15 per clinic
	HCW training manual	$21.33	Budgeted 5 per clinic
	Posters (clinics, community)	$3.64	Budgeted 10/clinic; plus, digital posters for BAs
	T-shirts for HCWs	$8.50	Budgeted 2 T-shirts/HCW, 6 HCW/clinic
Brand Ambassador	Ambassador toolkit/guide	$3.45	Budgeted 16–20 per clinic
	Ambassador certificates	$0.99	Budgeted 6 per clinic
	T-shirts for ambassadors	$8.50	Budgeted 2 T-shirts per BA, 2 BA per clinic
	Educational pamphlet	$0.22	Varied by clinic volume (use by BAs and HCW)
	Ambassador data bundle	$10.00	Budgeted per ambassador per month
	V parties (refreshment)	$1.00	$1/AGYW, $15/party (in-person only); 1 party/week
	V parties (airtime)	$1.00	$1/AGYW airtime for virtual attendance (online only)
AGYW Initiating PrEP *(Starter Kit)*	FAQ insert^	$0.91	Highly rated, universally well-liked IEC material
	Pill Case*^	$4.20	Highest rated among all V materials
	Makeup Bag*^	$2.35	Consider multiple color options
	Pill Box	$0.56	Consider dropping in scale up
	Reminder Sticker^	$0.15	Consider dropping in scale up
	Kitting*	$0.35	Cost to procure bundled kit (pill case, makeup bag)

*BA*  Brand Ambassador, *FAQ*  frequently asked questions, *HCW*  healthcare worker, *IEC*  information, education, and communication

^*^Procured for volume of 10,000 kits; ^#^Printed materials include 20% buffer for wastage

^Ranked most essential and useful by AGYW in Zimbabwe

## Discussion

### Acceptability and feasibility

Reducing new infections among AGYW requires addressing their large unmet HIV prevention needs by adapting strategies to fit their unique vulnerabilities. Early implementation learnings indicate “V” is an acceptable and feasible innovation to help support AGYW in engaging with PrEP as an HIV prevention strategy in Zimbabwe. “V” tackles many of the initiation-phase barriers to PrEP uptake as articulated by Rousseau et al. (2021) along a young woman’s PrEP user journey. V’s demand creation activities and peer outreach through Brand Ambassadors increases AGYW’s awareness about PrEP. V’s comprehensive educational materials and counseling on personal risk assessment improves AGYW’s perceptions of HIV risk and vulnerability.; V’s bold branding sparks curiosity and a discussion linking PrEP and self-care, reducing stigma or misconceptions about PrEP in the community. And V’s lip gloss shaped pill case for portability and discretion provides a practical solution for improving the ease of accessing PrEP through integrated services delivery models and community outreach sites for HIV testing, PrEP screening, and initiation [30].

In recent years innovative interventions to promote PrEP use among AGYW have been developed to address the unique experiences and needs of this population [31, 32]. “V” is a multi-level intervention in line with promising individual-, interpersonal-, community-, and health systems-level interventions including peer support for adherence [33], youth-friendly PrEP demand creation [34], de-medicalized PrEP delivery approaches [35], and PrEP integration with other services for AGYW [36, 37]. Although data on intervention effectiveness to improve PrEP uptake and continuation is currently limited, in part due to challenges with defining “successful” PrEP use among AGYW, many studies have demonstrated acceptability and feasibility [31]. Our evaluation builds on this existing literature and adds new insights to the evidence-base for multi-level interventions that concurrently address the complex and interrelated barriers facing AGYW, while also highlighting the critical role of HCD.

### Human-centered design

HCD helps translate innovative design principles that are resonant for addressing young women’s key PrEP barriers into contextually relevant interventions that are appealing to target users, while also highlighting what requires country specific adaptation to keep the innovative design principles culturally appropriate [26]. However, even after extensive immersion sessions with the target population and other key stakeholders, coupled with prototype feedback on the “V” assets through interactive stimuli and exercises, there were still ‘real world’ adaptations that emerged during the first few months of implementation. For example, several respondents mentioned unintended disclosure associated with “V” makeup bags, which had become associated with PrEP in some communities and prompted a suggestion to provide the makeup bag in a variety of colors going forward. On reflection, our team learned that a smaller scale “pre-pilot” or “soft launch” could have helped identify small tweaks or adaptations to materials prior to the full launch [38], or to recognize other factors, such as the widespread interest in the pill case among ART and PrEP users that required additional messaging from healthcare workers around the intervention’s focus on AGYW. Overall, these insights are in line with considerations around a phased approach from other HCD-based initiatives [39].

We learned that HCD can help support decision makers to determine whether and how to scale programming such as “V” by making sure we ‘get it right’ before going to scale [26, 34]. This work also inspires a number of additional questions in applying HCD to public health programming in the future, such as: How can we use HCD's agility to adapt to new challenges like COVID-19?; How can we use HCD to meet future PrEP needs, like increasing innovative financing models and private sector engagement, for example through private sector delivery outlets?; and as we realize these potential opportunities for HCD, what limitations do we need to keep in mind?

### Considerations for scale up

Learnings from this evaluation indicate “V” is acceptable in static and outreach sites implemented by private sector NGOs (PSI and PZAT), and that there could be broader applicability to integrate “V” within government facilities in Zimbabwe. Data on the effectiveness of “V” on PrEP uptake and continuation among AGYW will be available following one year of implementation and will be analyzed and synthesized alongside “V” early learnings. Scaling PrEP service availability through the public sector, including through outreach events, in all 62 districts of Zimbabwe would greatly expand access in the harder-to-reach rural areas, and “V” is a tool that could help support demand creation for PrEP among AGYW. Financing and sustainability are paramount to scale up considerations and we compiled the costs of “V” materials to help inform financial planning estimates of extending “V” to the public sector (under the assumption that donor and/or government resources could support scale up in the near term). Reducing costs through higher volume procurement as well as a customized lighter package of “V” materials, while still retaining V’s core approach, can be considered for public sector scaling. For example, dropping the makeup bag would reduce costs and lessen the potential for unwanted disclosure if the bag became associated with PrEP—an important consideration for scale up decisions given the seriousness of disclosure concerns. Additionally, recurrent costs, such as data costs for participation in online “V” parties, need further consideration as AGYW may be unlikely to self-sponsor to attend “V” parties. The design of online “V” parties can be revisited including the size and composition of these virtual events, which included both PrEP users and prospective PrEP users to facilitate greater sharing and learning. The Brand Ambassadors serve a critical function in running "V" parties, and more skills building on how to successfully facilitate virtual parties needs to be addressed in planning for scale.

Several additional health systems areas warrant further considerations for public sector scale up. First, programming needs to ensure sufficient support to developing Brand Ambassadors’ capabilities as effective change agents as “V” is scaled, given the social norm shifts required to achieve “V”’s reframing of oral PrEP as empowering self-care and whether there may be opportunities to build these skills in existing staffing cadres (e.g., younger community health workers to support in demand creation and peer support for "V"), and the type of training, coaching, and financial support necessary for implementation. Second, integrating training on PrEP and adolescent-friendly sexual and reproductive health service provision, for healthcare workers and community health workers. Third, supporting supply chain management to ensure a ready and continuous supply of PrEP and related tests, along with the selected “V” materials for scale up. And lastly, considering how to reach and maintain oral PrEP use among adolescent girls in schools, including further engagement with the Ministry of Primary and Secondary Education.

Alternate private sector models, such as engagement through pharmacies, malls, private clinics, and hospitals are worth further consideration and exploration [14, 40]. Young women in Zimbabwe had mixed reactions to the idea of accessing PrEP through alternate channels. Some expressed interest since it would eliminate the need to find a reason/excuse to get permission to visit a health facility, reduce the costs of travel, and is particularly applicable in the urban areas. However, other AGYW shared privacy concerns, for example if the pickup locations become associated with PrEP. Female sex workers were more interested in accessing PrEP through pharmacies and malls for the convenience factor of not needing to make an extra trip to the clinic for refills. In alignment with the original vision for “V”, these alternate private sector models could be explored further in the Zimbabwean context. For sustainability considerations, further studies should explore willingness to pay for “V” materials among the target users, the role of public–private partnerships to support scale up, and the frequency with which “V” branded materials may need refreshing to remain current with prevailing fashion trends among adolescents.

### Study limitations

Our study had several limitations that should be considered in interpreting the data. Due to the COVID-19 pandemic and increased safety precautions, all observations and interview data was collected virtually, which sometimes presented challenges due to dropped calls and limited the ability of the data collector to directly observe implementation fidelity. Because feasibility and acceptability data were collected only during the first three months of the intervention rollout, it may not be fully reflective of longer-term trends in these measures, and the short time frame also limited our ability to gain in-depth understanding of young women’s perspectives on PrEP continuation. Among young women interviewed, the sample was restricted to adults 18–24 years old and therefore did not gather perspectives from younger adolescents aged 15–17; however, the interviewer asked respondents to also reflect on their perspectives thinking back to earlier adolescence. In addition, current users of PrEP were overrepresented (73%) in the interview sample, in part because they were easier to recruit, which may have biased data toward stronger acceptability of V. Future program evaluation of “V” should ensure more balanced representation with AGYW who re-initiate, default, or choose never to use PrEP, and also consider prospectively following young women to explore changes in perspectives on “V” over a longer time horizon.

## Conclusion

After formative development in South Africa, the “V” brand and service delivery strategy was launched in Zimbabwe to reframe PrEP as an empowering self-care product that women desire. Site observations and interviews explored the acceptability of “V” and its relevance to target users, as well as the feasibility of integrating “V” with existing service delivery models. Through reframing PrEP, “V” demonstrates promise as a multi-level intervention to facilitate ease of access, uptake and continued use of PrEP among AGYW. Findings from this study add to a growing body of evidence on the feasibility and acceptability of multi-level interventions to improve PrEP access, uptake, and continuation among AGYW, and can be further adapted to suit a variety of country contexts. Policy makers and programmers are encouraged to consider higher volume procurement and a customized lighter package of “V” materials, which can retain V’s core approach while promoting broader scaling.

### Supplementary Information

**Additional** **file** **1.** Site Monitoring Checklist.

**Additional** **file** **2.** Interview Guide.

## Data Availability

The qualitative datasets generated during the current study are not publicly available as consent did not include publishing the dataset publicly; however, data are available from the corresponding author on reasonable request.

## Abbreviations

AGYW: Adolescent girls and young women
ART: Antiretroviral therapy
BA: Brand Ambassador
FAQ: Frequently asked questions
HCD: Human centered design
HCW: Health care worker
HIV: Human immunodeficiency virus
IEC: Information, education, and communication
MoHCC: Ministry of Health and Child Care
NSC: New Start Center
PEPFAR: President’s Emergency Plan for AIDS Relief
PrEP: Pre-exposure prophylaxis
PSI: Population Services International
PZAT: Pangaea Zimbabwe AIDS Trust
SHAZ!: Shaping the Health of Adolescents in Zimbabwe
SMS: Short message service

## Footnotes

1 Design in global health spans a range of definitions that is beyond the scope of this review to discuss in full. Defined by the Design for Health community as “a craft and a discipline that applies a specific process, mindset and skill set to a creative problem-solving process,” terms such as HCD, design thinking and experience design have in common a process that prioritizes direct engagement with a wide range of stakeholders, a skillset that values creative facilitation approaches to encourage co-creation via iterative prototyping, and an empathy-focused mindset.
