# Supplementary material for: Early implementation learnings on acceptability and feasibility of “V”: a multi-level PrEP intervention designed with and for adolescent girls and young women in Zimbabwe
Source: Health Res Policy Syst. 2023 Oct 2;21:101. doi: 10.1186/s12961-023-01040-3 (PMC10546763; doi:10.1186/s12961-023-01040-3)
Supplement: Supplementary file 1 — Additional file 1. Site Monitoring Checklist. [file 12961_2023_1040_MOESM1_ESM.docx]

**Site Monitoring Checklist**

**Accelerating uptake and continuation of Oral PrEP through “V” in Zimbabwe**

This site monitoring checklist is used to capture the information regarding PrEP service provision at the facility that is participating in the V Pilot project. It assesses the extent to which all the elements required for successful implementation of V are present during the pilot. This checklist will be administered three times at different intervals.

| **Date of Site Visit** |  |
| --- | --- |
| **Name of Facility** |  |
| **Dates trained on “V”** |  |
| **Date stated implementing “V”** |  |
| **Name of Interviewer** |  |
| **Name (s) of Interviewee(s)** |  |

| **NO** | **QUESTION** | | **OBSERVATION NOTES** | |
| --- | --- | --- | --- | --- |
| **SECTION 1. PrEP STATISTICS** | | | | |
| **SECTION 2. HUMAN RESOURCES** | | | | |
| **2** | **For each of the healthcare worker (HCW) cadres below, indicate the number at the facility, how many were trained on Oral PrEP, and how many were trained on “V”?** | | | |
|  | **HCW Cadre** | **Total at facility** | **Trained on Oral PrEP (Standard MoHCC)** | **Trained on “V”** |
|  | Medical Doctor |  |  |  |
|  | Clinical Officer |  |  |  |
|  | Registered General Nurse |  |  |  |
|  | Primary Care Nurse |  |  |  |
|  | Primary Counsellor |  |  |  |
|  | Medical Lab Scientist |  |  |  |
|  | Pharmacist |  |  |  |
|  | Pharmacy Assistant |  |  |  |
|  | Other(s) |  |  |  |
| **2.1** | In general, who is doing PrEP work in this facility? | | | |
| **2.2** | Please give a brief description on how your facility is currently doing demand creation for PrEP using V. *(Integration of V into the existing system)* | | | |
| **3** | **Are there HCW trained on “V” at the clinic today?** | | | |
|  | Yes🞏 How many HCW(s):_____________________ | |  | |
|  | No 🞏 State reason: __________________________ | |  | |
| **4** | **For each of the community-based cadres below, indicate the number linked with the facility, how many were trained on Oral PrEP, and how many were trained on “V”?** | | | |
|  | **Community Cadre** | **Total linked w/facility** | **Educated on PrEP** | **Trained on “V”** |
|  | Brand ambassadors (PSI) |  |  |  |
|  | PrEP champions (PSI, PZAT) |  |  |  |
|  | Community mobilizers (PZAT) |  |  |  |
|  | Other, specify: ____________ |  |  |  |
|  | Other, specify: ____________ |  |  |  |
|  | Other, specify: ____________ |  |  |  |
|  | Other, specify: ____________ |  |  |  |
| **5** | **Are there community-based cadres, with training on “V”, observable during the site visit?** | | | |
|  | Yes 🞏 | |  | |
|  | No 🞏 State reason(s): ________________________ | |  | |
| **SECTION 3. COMMODITIES** | | | | |
| **6** | **Are “V” Starter Kits available at the facility?** | | | |
|  | Yes 🞏 *Approximately how kits many on hand: _____* | |  | |
|  | No 🞏 *Why not:* ________________________­­­­____ | |  | |
| **7** | **Are PrEP screening forms available at the facility?** | | | |
|  | Yes 🞏 | |  | |
|  | No 🞏 State reason(s):________________________ | |  | |
| **8** | **Are HIV rapid tests kits available at the facility?** | | | |
|  | Yes 🞏 *Stock on hand:* | | Note: check the stock card to work out the months of stock available | |
|  | No 🞏 State reason(s):________________________ | |  | |
| **9** | **Are menstrual hygiene products available at the facility?** | | | |
|  | Yes 🞏 *Stock on hand:* | | Type of products and quantity  1.  2.  3.  .  .  x | |
|  | No 🞏 State reason(s):________________________ | |  | |
| **10** | **Are pregnancy tests available at the facility?** | |  | |
|  | Yes 🞏 *Stock on hand:* | |  | |
|  | No 🞏 State reason(s):________________________ | |  | |
| **11** | **Is there adequate PrEP drug stock (TDF/FTC) available at the facility?** | | | |
|  | Yes 🞏 *Stock on hand:* | | Note: check the stock card to work out the months of stock available | |
|  | No 🞏 State reason(s):________________________ | |  | |
| **12** | **Has this facility experienced a stock out of PrEP drugs (TDF/FTC) in the last 3 months?** | | | |
|  | Yes 🞏 State reason(s):________________________ | |  | |
|  | No 🞏 | |  | |
| **SECTION 4. FACILITY-BASED SERVICE DELIVERY *(Fidelity to Implementation of V)*** | | | | |
| **13** | **During the process of interaction at all service points, is the nurse referring AGYW clients for HIV counseling and testing?** | | | |
|  | Yes 🞏 | |  | |
|  | No 🞏 State reason(s):________________________ | |  | |
| **14** | **Which departments are administering the PrEP screening form to AGYW in the facility?*)***  *(Check all that apply)* | | | |
|  | HIV counseling & testing 🞏  STI screening 🞏  Family Planning 🞏  ART 🞏  OPD 🞏  Other 🞏 | |  | |
|  | No, not being administered to AGYW 🞏 | | State reason(s):________________________ | |
| **15** | **Is there a clear entry point for administration of the PrEP screening form at the facility?** | | | |
|  | Yes 🞏 | |  | |
|  | No 🞏 State reason(s):________________________ | |  | |
| **16** | **Are V posters and educational materials visible to patients attending the health facility?** | | | |
|  | Yes 🞏 Where placed? | |  | |
|  | No 🞏 State reason(s):________________________ | |  | |
| **17** | **Are AGYW that test negative for HIV (rapid or self-test) subsequently referred for PrEP screening?** | | | |
|  | Yes 🞏 | |  | |
|  | No 🞏 State reason(s):________________________ | |  | |
| **18** | **Are AGYW offered a pregnancy test before proceeding to PrEP initiation?** | | | |
|  | Yes 🞏 | |  | |
|  | No 🞏 State reason(s):________________________ | |  | |
| **19** | **If an AGYW is eligible for PrEP, does the HCW administer the PrEP readiness assessment?** | | | |
|  | Yes 🞏 | |  | |
|  | No 🞏 State reason(s):________________________ | |  | |
| **20** | **Are HCW observed actively using the V reference materials to discuss PrEP with AGYW?** | | | |
|  | Yes 🞏 | |  | |
|  | No 🞏 State reason(s):________________________ | |  | |
| **21** | **Which V reference materials do the HCWs utilize?** *List/describe.* | | | |
| **22** | **At what point in the PrEP discussion are HCW observed using V materials and/or discussing V?** *Describe.* | | | |
| **23** | **Are eligible and consenting AGYW initiated on PrEP?** | | | |
|  | Yes 🞏 | |  | |
|  | No 🞏 State reason(s):________________________ | |  | |
| **24** | **Are “V” Starter Kits being distributed to AGYW when initiating PrEP?** | | | |
|  | Yes 🞏 | |  | |
|  | No 🞏 State reason(s):________________________ | |  | |
| **25** | **Where do AGWY receive their 1^st^ month of PrEP drugs?** | | | |
|  | From the nurse when receiving the V starter kit 🞏  From the pharmacy after receiving the V starter kit 🞏  From some other location 🞏 Specify: _____________ | |  | |
|  | No drugs received 🞏 State reason(s):____________ | |  | |
| **26** | **Are HCW friendly, respectful and non-judgmental in offering PrEP to AGYW?** | | | |
|  | Yes 🞏 | |  | |
|  | No 🞏 | |  | |
| **27** | **Are discrete (private) HIV services accessible and convenient for AGYW at this facility?** | | | |
|  | Yes 🞏 Describe. | |  | |
|  | No 🞏 Describe why not: | |  | |
| **SECTION 5. OUTREACH-BASED SERVICE DELIVERY *(Fidelity to Implementation of V)*** | | | | |
| **28** | During provision of outreach services other than HTS, is the offering AGYW clients HIV counselling and testing services? | | | |
|  | Yes 🞏 | |  | |
|  | No 🞏 State reason(s):________________________ | |  | |
| **29** | **At what point during outreach do HCW administer the PrEP screening form to AGYW?**  *(Check all that apply)* | | | |
|  | HIV counseling & testing 🞏  Family Planning 🞏  Other 🞏 | |  | |
|  | No, not being administered to AGYW 🞏 | |  | |
| **30** | **Do community-based cadres administer the PrEP screening form?** | | | |
|  | Yes 🞏 Where and when: ______________________ | |  | |
|  | No 🞏 State reason(s):________________________ | |  | |
| **31** | **If a AGYW is eligible for PrEP, does the HCW administer the PrEP readiness assessment at the community outreach site?** | | | |
|  | Yes 🞏 | |  | |
|  | No 🞏 State reason(s):________________________ | |  | |
| **32** | **Are AGYW offered a pregnancy test before proceeding to PrEP initiation at community outreach site?** | | | |
|  | Yes 🞏 | |  | |
|  | No 🞏 State reason(s):________________________ | |  | |
| **33** | **Are HCW observed actively using the V reference materials to discuss PrEP with AGYW during community outreach visits?** | | | |
|  | Yes 🞏 | |  | |
|  | No 🞏 State reason(s):________________________ | |  | |
| **34** | **Which V reference materials do the HCWs utilize during community outreach?** *List/describe.* | | | |
| **35** | **At what point in the PrEP discussion are HCW observed using V materials and/or discussing V?** *Describe.* | | | |
| **36** | **Are eligible and consenting AGYW initiated on PrEP at the community outreach site or referred to the clinic for initiation?** | | | |
|  | Yes, initiated at outreach site 🞏 | |  | |
|  | No, referred to clinic for initiation 🞏 | |  | |
| **37** | **Are “V” Starter Kits being distributed to AGYW when initiating PrEP at community outreach site?** | | | |
|  | Yes 🞏 | |  | |
|  | No 🞏 State reason(s):________________________ | |  | |
| **38** | **Where do AGWY receive their 1^st^ month of PrEP drugs when initiating during outreach visits?** | | | |
|  | From the nurse during the community outreach visit 🞏  Must return to the clinic pharmacy for PrEP drugs 🞏  From some other location 🞏 Specify: _____________ | |  | |
| **39** | **Are discrete (private) HIV services accessible and convenient for AGYW in the outreach setting?** | | | |
|  | Yes 🞏 Describe _____________________________ | |  | |
|  | No 🞏 Describe why not:______________________ | |  | |
| **SECTION 6: COMMUNITY-BASED SERVICES *(Fidelity to Implementation of V)*** | | | | |
| **40** | **Are trained CHW using V reference materials when offering demand generation services?** | | | |
|  | Yes 🞏 Which materials? _______________________ | |  | |
|  | No 🞏 Why not? _____________________________ | |  | |
| **41** | **Are trained ambassadors using V reference materials when offering demand generation services?** | | | |
|  | Yes 🞏 Which materials? _______________________ | |  | |
|  | No 🞏 Why not? _____________________________ | |  | |
| **42** | **Are ambassadors undertaking patient follow-up for AGYW identified in the community who may be eligible for PrEP? (e.g. for AGYW they may still be considering PrEP)** | | | |
|  | Yes 🞏 State follow up methods: _________________ | |  | |
|  | No 🞏 Why not? | |  | |
| **43** | **Are ambassadors undertaking patient follow-up for AGYW who initiated PrEP to provide support for continuation etc.?** | | | |
|  | Yes 🞏 State follow up methods: _________________ | |  | |
|  | No 🞏 Why not? | |  | |
| **44** | **Are ambassadors sending weekly reminder SMS to AGYW on PrEP who want reminders?** | | | |
|  | Yes 🞏 How referring to V? _____________________ | |  | |
|  | No 🞏 Why not? _____________________________ | |  | |
| **45** | **Have V ambassador gatherings have been conducted to-date?** | | | |
|  | Yes 🞏 Number of gatherings: ___________________ | | Type of gatherings: _________________ | |
|  | No 🞏 Why not? _____________________________ | |  | |
| **SECTION 7: MONITORING AND EVALUATION** | | | | |
| **46** | **Are standard procedures and systems for tracking and reporting uptake of PrEP delivery being applied?** | | | |
|  | Yes 🞏 | |  | |
|  | No 🞏 | |  | |
| **47** | **Is reporting of PrEP indicators linked to the national DHIS2?** | | | |
|  | Yes 🞏 | |  | |
|  | No 🞏 | |  | |
| **SECTION 8: EMERGING ISSUES** | | | | |
| **48** |  | | | |
| **SECTION 9: RECOMMENDATIONS FOR IMPROVING “V” IMPLEMENTATION** | | | | |
| **49** |  | | | |
| **50** | **Please may you show me around the facility in live video.** (Record all V – materials observed) | | | |
