# Supplementary material for: Early implementation learnings on acceptability and feasibility of “V”: a multi-level PrEP intervention designed with and for adolescent girls and young women in Zimbabwe
Source: Health Res Policy Syst. 2023 Oct 2;21:101. doi: 10.1186/s12961-023-01040-3 (PMC10546763; doi:10.1186/s12961-023-01040-3)
Supplement: Supplementary file 2 — Additional file 2. Interview Guide. [file 12961_2023_1040_MOESM2_ESM.docx]

**INTERVIEW GUIDE**

**Health Care Workers**

Date of Interview: ___/____/2021

**Did you participate in Round 1 Interviews? Yes/No.**

**PART 1: DEMOGRAPHICS** (Basic Facts)

| **Name of Study Site** | Chipinge NSC | Gweru NSC | Bulawayo NSC | SHAZ Hub |
| --- | --- | --- | --- | --- |
|  |  |  |  |  |
| **Age** |  | | | |
| **Gender** |  | | | |
| **Job Title** |  | | | |
| **Qualifications** |  | | | |
| **Summary description of Respondent’s Role** |  | | | |
| **Length of Time in Study Site** |  | | | |
| **Start Date Working on Implementation of V** |  | | | |
| **How have you been working on V? Your role and how were others complementing you?** |  | | | |

**PART 2:**

**A. GENERAL INFORMATION ABOUT PREP.**

1. **Please describe what V is and what V aims to achieve.**
   - What is your understanding of how V is intended to be implemented? (e.g., implementing V per the training plans and as designed)
2. **In general, how is the V Campaign going?**
   - Are there any adaptations you have made in your work routine that you had to make to accommodate V? What are they and why did you have to make these? After making these adaptations how is work flowing? *(Reassure the participant there are no right or wrong answers: For V, we are trying to learn what is working well and working less well may need site level modifications).*
3. **What is the general profile of PrEP users at your clinic?**
   - Are these any different from the ones you had before V was introduced?
   - Are they all coming from the NSC catchment area or they come from other Zones
   - What are the motivations for these clients to use PrEP?
     - - 1. How do you think V is influencing decision making, if at all?
         2. Is it to take PrEP or just to have the V assets? Why do you think so?
         3. Are there any AGYW who have been offered V materials and refused to take them? Or they decided to take some of the assets and not others? If yes, which did they exclude and what were their reasons?
   - Thinking back to before V was introduced: Among PrEP-eligible AGYW who know about PrEP and refuse to use PrEP: What are their reasons?
   - Now that V has been introduced: Among PrEP-eligible AGYW who know about PrEP and V but refuse to use PrEP? What are their reasons?
4. **Barriers: When you think of an AGYW coming to the clinic and outreach sites, what do you feel are the barriers for accessing PrEP?**
   - Of these which one do you think is the most significant?
   - Is V able to address any of the barriers? How/why? (be specific!)
5. **What is the role of CHW (e.g. brand ambassadors, CATS etc.) in demand creation for PrEP?**
   - How are they supporting demand generation as well as adherence and continuation on PrEP?
   - How is that working so far? Are there any challenges and opportunities?
   - Have they provided you with any feedback on their experience in utilizing V assets in demand creation and/or in supporting AGYW with continuation? If yes, please share the feedback…

| *“V” promotes more positive relationships with healthcare workers and friendlier clinic environments by providing* ***specific resources to transform the clinical experience and empower healthcare workers*** *with resources designed to resonate with a young female audience. “V” was recently launched in this clinic and in the next set of questions, we want to get your perspectives on implementing “V” within your healthcare setting – including barriers and facilitators to implementing “V”, the feasibility and acceptability of implementing “V”, and overall input on improvements going forward.* |
| --- |

**B. “V” MATERIALS – HEALTH CARE WORKERS**

1. **Which V materials have you used in your clinic?**
   1. How have you used each of these materials? [note: don’t ask about each asset specifically – we are interested in assets brought up by memory as the ones they use, not that they are able to list all of them]
2. **Which of these materials have worked well in helping you, if at all, to engage and interact with AGYW at the clinic and during outreach to learn more about PrEP? Which ones have not worked well? Why you think so?**
   1. For each of the assets you mentioned above -- which pages or what specific content are most helpful?
      1. How did they help you to provide counselling and create interest in starting on PrEP (or re-starting)? *i.e., helping the girls to assess their risk, consider and decide to take PrEP.*
      2. How did they help you to prepare the client for PrEP? *i.e., getting the clients ready to take PrEP as a daily dose, what to expect (side effects, myths and misconceptions etc.)*
      3. How did they help you to encourage AGYW to take a pill every day?
         1. How are the V assets helping to support habit formation/reminder cues to take pills on a regular schedule?
         2. Which of the V assets are they using as reminder cues?
         3. How are they using them?
   2. Which of the assets would you say is a ‘game changer’? Why?
   3. Are there materials that you are not using at all? Why are you not utilizing them?
      1. Probe whether there was an issue with training in some way – and if refresher training may be needed.
3. **Are there any differences in how you are engaging and interacting with AGYW before and after the introduction of V? please explain.**
4. **Has “V” been effective in creating more demand for PrEP among AGYW? Why or why not?**
   1. Are clients satisfied with the “V” materials? Which ones?
   2. Do you think they like the materials? Why or why not?
   3. Which of the materials appear to generate the most happiness for the AGYW? Why?
   4. Are they using the assets for what they are intended for?
      1. Probe – bag, pill-case. box, sticker
      2. What else are they using them for?
5. **Has “V” been effective in improving PrEP continuation among AGYW? Why or why not?**
   1. Are there any aspects of the delivery of “V” that could be modified to further promote continuation?
      1. Probe on sticker, pill-case
      2. What else do they use for reminders?
      3. Probe on refill pick up at non-traditional locations (private sector pharmacies, community distribution for refills, others?)
   2. Are there AGYW who have discontinued PrEP after they received the V starter kits?
      1. What are their reasons for discontinuing? Are the reasons linked to V? Why?
      2. How did they feel about the contribution of V to their user experience for PrEP?
      3. What in V could help them to restart and stay on PrEP?
   3. Based on your experience comparing before and after V was introduced: have you noticed any changes in the number of girls initiating PrEP that are returning for their 1-month refill?
6. **Does your clinic currently integrate PrEP offerings with any other services, for example, family planning? Why or why not?**
   1. Describe how this integration has been done?
   2. How is this integration working?
   3. Do you have any suggestions on how we can improve the clinic flow for integrating V into services for AGYW? What could be done differently?
7. **Do you think “V” is feasible, for HCW to continue implementing over a long time as a strategy for improving PrEP initiation and continuation among young women?**
   1. What would change in the absence of V?
   2. Do you think this same approach can be used in the government public facilities? Why? Why Not?
   3. How can this approach be integrated into the current PrEP delivery system in Zimbabwe.
   4. Do you think AGYW would prefer to collect their PrEP refills at the pharmacies nearest to them? Where else could they prefer to? Why?
8. **Are there any un-intended consequences associated with V that you know of? How have you dealt with them?**
   1. What are the guardians/parents of AG and general population saying about V?
   2. What are the negative things you have heard about V so far?
   3. What are the reasons why people say or think so about V?
9. **Are there any challenges you have faced in service delivery as a result of the introduction of V?**
   1. How have you worked around the challenges?
   2. Did V result in additional work within your routine service provision? If yes, how are you managing this additional workload for initiating AGYW?
10. **What additional support do healthcare providers at your clinic or in the community need to implement “V” successfully? Any further modifications or improvements needed?**
    1. Do you have any suggestions on how we can improve the V materials being used in this clinic and on outreach? Why? What are your suggestions for improving these?
11. **What additional updates, if any, to clinic flow and operations are needed to support the use of the V materials? [**e.g. changes to ensure confidentiality, clinic operating hours and days, mobile operations, outreach via CHWs…]
12. **Is there anything further that we can share about the "V" project? Opportunities?**

**-END-**

**INTERVIEW GUIDE**

**Community Health Workers (Brand Ambassadors)**

Date of Interview: ___/____/2021

**Did you participate in Round 1 Interviews? Yes/No.**

**PART 1: DEMOGRAPHICS** (Basic Facts)

| **Name of Study Site** | Chipinge NSC | Gweru NSC | | Bulawayo NSC | SHAZ Hub |
| --- | --- | --- | --- | --- | --- |
|  |  |  | |  |  |
| **Job Title of Respondent** |  | | | | |
| **Age** |  | | | | |
| **PrEP Use** | Yes – Current User | |  | | |
|  | Yes – Previous User | |  | | |
|  | No – Never | |  | | |
|  | Prefer not to disclose | |  | | |
| **Summary description of Respondent’s Role** |  | | | | |
| **Length of Time attached to this Study Site** |  | | | | |
| **Start Date Working on Implementation of V** |  | | | | |
| **Are there any other jobs you do outside the BA roles?** |  | | | | |
| **How Many V- Parties Have you held since the start of V?** |  | | | | |

1. **What is your PrEP client profile?**
2. Age, what they do, where you usually find them?
3. What do they think generally about PrEP
4. **What is V?**
   1. How is V implemented based on your understanding from the training?
   2. How has implementation been so far?
5. **Please describe your role in educating AGYW about HIV prevention, and PrEP specifically (Using V)?**
   1. Have you been trained on V Campaign?
   2. How do you work with AGYW? Describe how you have been able to identify and engage with them (using V?)
   3. How has V changed the way you engage the AGYW?
   4. How has your approach to engaging AGYW changed during COVID-19, if at all?
6. **What are the barriers and motivations for AGYW to access PrEP?**
7. Of these, which one do you see as the most significant?
8. How is V influencing (if at all) the motivations and barriers to AGYW to access PrEP?
9. How has your approach to engaging AGYW changed since we introduced V, if at all?
10. **Which V materials have you used (if any) and how have you used them? Probe: Name of material and how it is used to:**
    1. Which materials have been most useful for you in:
       - Explaining the basics of oral PrEP and its benefits? (Awareness and knowledge)
       - Creating demand
       - Explaining where to get oral PrEP? (Accessibility and availability)
       - Improving accuracy of risk perception among AGYW
       - Improving knowledge about PrEP and supporting continuation
    2. How have the materials changed how you interact with AGYW about PrEP? Is this the same for physical and online engagements?
       1. Which materials have stirred strong engagement online? Physical?
    3. Are there materials that you are not using at all? Why not?
11. **Have you facilitated any V-Parties?**
    1. Please take me through the process you undertake when preparing for the v-parties, (from planning to actual undertaking of the party)
       1. *Probe on how V is integrated into community mobilization and crowd pulling.*
    2. How many have you done so far?
    3. When did you begin holding the V Parties?
    4. On average how many AGYW would attend a session?
    5. How long would each session last in general?
    6. What are the FAQ during the V parties?
    7. Have you been able to respond to those questions using V materials?
    8. Probe on any differences with the previous experience with Ambassador gatherings and how they have blended the two approaches
12. **How has the V-Approach changed/influenced your BA experience in promoting PrEP continuation?**
    1. In your opinion, do you think V is successful in both promoting continuation?
    2. Please describe the activities you undertake in promoting continuation using V.
13. **Do you think V is contributing to the AGYW confidence to take PrEP and remain HIV-?**
    1. How about Empowerment *e.g. being able to talk about PrEP and why they are taking it?*
    2. How about Taking Control *e.g. being in charge of maintaining the HIV status negative and not relying on the cooperation of their partners for HIV prevention such as using the condoms? How/Why*
14. **Do you think V has empowered the AGYW to disclose their PrEP use to friends and partners? How/Why.**
    1. Which specific material have contributed to this e.g. Pill case – Empowerment and discretion?
    2. Are there some AGYW you know who have disclosed that they are taking PrEP to their partners/parents/guardians?
    3. What are the experiences they had after they disclosed?
    4. What do you think motivated them to do so?
    5. How has the whole starter kit or the elements of the starter kit (FAQs, Pill case, Sticker, box, bag) helped the AGYW talk with partners/family/friends about HIV prevention and PrEP.
    6. On the FAQ booklet, which pages are most useful? (e.g., the myth busters, side effects)
    7. What about those who have not yet disclosed? What are their reasons?
    8. What do other people say about V? Why do they say this? Probe to assess if there is any stigma around V assets?
15. **Do you think “V” is a feasible strategy to use for a long time for improving PrEP initiation and continuation among AGYW? Why or why not?**
    1. Do you think this same approach can be used in the government public facilities? Why? Why Not?
    2. How can this approach be integrated into the current PrEP delivery system in Zimbabwe?
    3. Do you think AGYW would prefer to collect their PrEP refills at the pharmacies nearest to them? Where else could they prefer to? Why?
16. **Are there any un-intended consequences associated with V that you know of?**
    1. How have you dealt with them?
17. **What additional support do you CHWs need to implement “V” successfully? Any further modifications or improvements needed, particularly in the times of COVID-19?**
    1. What challenges have you faced with V? How have you worked around this?
    2. Do you have any suggestions on how we can improve the materials being used by CHWs in your area?
18. **Is there anything further that we can share about ourselves or the “V” project?**

**END**

**INTERVIEW GUIDE**

**YOUNG WOMEN (V Users)**

Date of Interview: ___/____/2021

**PART 1: DEMOGRAPHICS** (Basic Facts)

| **Name of Study Site** | Chipinge NSC | Gweru NSC | | | Bulawayo NSC | SHAZ Hub |
| --- | --- | --- | --- | --- | --- | --- |
|  |  |  | | |  |  |
| Age of Participant |  | | | | | |
| Client Group Classification (Circle) | GP (general pop), FSW, TSW, BS, WSW, MSM | | | | | |
| Marital status (circle) | Cohabitating | |  | Divorced | |  |
|  | Married | |  | Never Married | |  |
|  | Separated | |  | Widowed | |  |
| Educational status (circle) | Primary | |  | Secondary | |  |
|  | Tertiary | |  | None | |  |
| PrEP User Status | Yes – Current User | |  |  | | |
|  | Yes – Previous User | |  |  | | |
|  | Prefer not to disclose | |  |  | | |
| Length of Time on PrEP / Used PrEP before discontinuing |  | | | | | |
| Length of time using V assets |  | | | | | |
| How long have you lived in this area? |  | | | | | |
| Is this your usual health facility where you seek health services? |  | | | | | |

1. **How long have you been using PrEP?**
2. **Can you tell me about how you’ve used PrEP during this last week? (keep this open ended, then ask questions below if participant does not raise them naturally.)**

- What time do you usually take PrEP?
- Why did you choose this time?
- Do you ever forget to take your PrEP?

1. **Have you heard of “V”?**
   1. Where did you first learn about “V”? From whom?
   2. Imagine you are explaining V to a family member, friend or partner. Pretend I’m that person and tell me about “V” in your own words?
2. **Were you issued a V starter kit?**
   1. When and where did you receive the starter kit from? (Static clinic or outreach?)
   2. What did you receive as part of the starter kit?
   3. Did you have any questions about the starter kit and how to use it/PrEP?
   4. Were those questions answered?
   5. Did you start using it immediately?
3. **How are you using the V starter kit?**
   1. What do you think about the starter kit?
   2. Did the starter kit motivate you to start taking PrEP?
   3. How are you using each component (bag, pill case, FAQ booklet, sticker, box)?
   4. Is this how you were taught to use it? If not, why did you choose to use it your way?
   5. Which components of the V starter kit are your favorites? Why?
   6. If you could only keep one thing from the starter kit, what would it be?
4. **How is V helping you with continuation? You have been on PrEP for (*X weeks – as per answer provided earlier),* how has V helped you keep taking your PrEP? Probe:**
   1. How do you make sure you don’t forget to take PrEP? Probe
      - Reminders (pill case, sticker, FAQ booklet)
      - What else do you do to remember to take your PrEP?
   2. How does V help you keep your PrEP use private/discreet?
      - Probe: Box, pill case, bag
      - *(Note: If only recently began taking PrEP: reframe as, how do you think V will be helpful in reminding you to continue taking PrEP? Why?)*
   3. How easy has it been for you to adhere to PrEP?
      - Do you feel confident that you can continue taking your PrEP as directed and without gaps in use?
   4. Do you feel confident and in control of your maintaining your negative HIV status? Why?
      - Has V helped you feel more confident or in control? Please explain.
   5. Have you used PrEP continuously since the time you began using PrEP? If not, probe, why they stopped and what motivated them to re-start.
5. **Overall, what do you think about “V”? Probe on:**
   1. How does V make you feel in general?
   2. How did/does V make you feel about your ability to prevent HIV?
   3. Delight: Does V make you happy (or make you smile)? Why?
6. **Why did you choose to take PrEP? Probe**
   1. How did V help you to make this decision? (Probe on content in IEC materials; improving your risk perception and confidence you can stay HIV negative with PrEP/V; information about PrEP, HIV, how to manage side effects)
   2. If the V starter kit had not been offered to you, would you have taken PrEP? Why/why not?
7. **Disclosure about PrEP/V**
   1. Have you told a family member, friend or partner about your PrEP use in the past? How did they react to it?
   2. Since receiving the V starter kit, have you told anyone about your PrEP use? If yes, pretend I’m that person and tell me what you said. Did you use any of the components of the V starter kit as “props” during that conversation?
   3. Does having the V starter kit make you feel more/less confident about disclosing your PrEP use? Please explain.
8. **Do you have friends that use “V”? What do they say about it?**
   1. Do any of your younger friends use “V” e.g. Under 18s?
   2. What do they say about it? What do they like or dislike about it?
   3. Do you think “V” could encourage you or someone else to start PrEP? Probe:
      - information for realistic risk perception
      - convenience of taking PrEP using V pill-case as both a reminder and discreet tool
      - confidence that V will help empower one to stay negative
   4. Thinking back to your teenage years, would “V” have been an attractive option for you? Why or Why not?
9. **Have you attended any V-parties? If yes,**
   1. How was your experience at the party? What did or did you not enjoy?
   2. Did you learn any information at the party that you didn’t know before? Probe:
      - Realistic risk assessment
      - PrEP as HIV prevention
      - PrEP side effect, myths, misconceptions
      - Continuation support (e.g., reminders, WhatsApp groups, etc.)
      - Deciding whether or not to disclose
10. **Have you received any support on continuation on PrEP from the brand ambassadors and health care workers? Probe: What kind of support?**
    1. Are you on the Let’s Talk WhatsApp Groups?
    2. How often does the BA send messages about V?
    3. What do you chat about?
    4. What is helpful about this support?
    5. Is there anything that could be better?
11. **Where did you get your first PrEP supplies? Have you consistently obtained your supply from the same place? … why or why not?**
    - Do you like receiving your supplies the way you are getting them now?
    - What other options would you prefer? Probe: Pharmacies? Dispensing Machines? Mail? Government Facilities?
    - What if PrEP could be bundled with other products such as menstrual products, would that be desirable to you? Please explain.
12. **Where did you first initiate on PrEP? What could have been better about it?**
    1. If you could have initiated on PrEP without going to a clinic, would that be desirable to you? Please explain.
    2. If you could have answered questions about your level of risk via an online survey, a private WhatsApp chat or over the phone with a nurse/counselor, would that have been of interest to you? Please explain.
13. **Is there anything else that you’d like to share with us about V?**

**END**
